# Isopropyl 2-(4,6-dimethyl-3-methyl­sulfinyl-1-benzofuran-2-yl)acetate

**DOI:** 10.1107/S1600536808034466

**Published:** 2008-10-25

**Authors:** Hong Dae Choi, Pil Ja Seo, Byeng Wha Son, Uk Lee

**Affiliations:** a Department of Chemistry, Dongeui University, San 24 Kaya-dong, Busanjin-gu, Busan 614-714, Republic of Korea; b Department of Chemistry, Pukyong National University, 599-1 Daeyeon 3-dong, Nam-gu, Busan 608-737, Republic of Korea

## Abstract

Mol­ecules of title compound, C_16_H_20_O_4_S, which was synthesized by the oxidation of isopropyl 2-(4,6-dimethyl-3-methyl­sulfanyl-1-benzofuran-2-yl)acetate, inter­act through C—H⋯π inter­actions between a methyl­ene H atom and the aromatic carbon ring of the benzofuran ring system, and by C—H⋯O hydrogen bonds. Adjacent stacked mol­ecules exhibit a carbon­yl–carbonyl inter­action [3.295 (2) Å]. The O atom of the methyl­sulfinyl group is disordered over two positions with site-occupancy factors of 0.9 and 0.1.

## Related literature

For the crystal structures of similar alkyl 2-(3-methyl­sulfinyl-1-benzofuran- 2-yl)acetate derivatives, see: Choi *et al.* (2007 ▶, 2008 ▶). For a review of carbon­yl–carbonyl inter­actions, see: Allen *et al.* (1998 ▶).
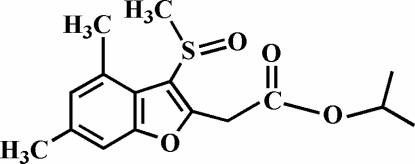


## Experimental

### 

#### Crystal data

C_16_H_20_O_4_S
*M* *_r_* = 308.38
Triclinic, 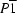

*a* = 6.308 (1) Å
*b* = 11.340 (2) Å
*c* = 11.506 (2) Å
α = 81.403 (3)°
β = 77.205 (3)°
γ = 83.167 (4)°
*V* = 790.4 (2) Å^3^
*Z* = 2
Mo *K*α radiation
μ = 0.22 mm^−1^
*T* = 298 (2) K
0.40 × 0.30 × 0.10 mm

#### Data collection

Bruker SMART CCD diffractometer
Absorption correction: none
4195 measured reflections
2760 independent reflections
2270 reflections with *I* > 2σ(*I*)
*R*_int_ = 0.044

#### Refinement

*R*[*F* ^2^ > 2σ(*F* ^2^)] = 0.043
*wR*(*F* ^2^) = 0.126
*S* = 1.05
2760 reflections
202 parameters
7 restraints
H-atom parameters constrained
Δρ_max_ = 0.40 e Å^−3^
Δρ_min_ = −0.27 e Å^−3^

### 

Data collection: *SMART* (Bruker, 2001 ▶); cell refinement: *SAINT* (Bruker, 2001 ▶); data reduction: *SAINT*; program(s) used to solve structure: *SHELXS97* (Sheldrick, 2008 ▶); program(s) used to refine structure: *SHELXL97* (Sheldrick, 2008 ▶); molecular graphics: *ORTEP-3* (Farrugia, 1997 ▶) and *DIAMOND* (Brandenburg, 1998 ▶); software used to prepare material for publication: *SHELXL97*.

## Supplementary Material

Crystal structure: contains datablocks global, I. DOI: 10.1107/S1600536808034466/ng2503sup1.cif

Structure factors: contains datablocks I. DOI: 10.1107/S1600536808034466/ng2503Isup2.hkl

Additional supplementary materials:  crystallographic information; 3D view; checkCIF report

## Figures and Tables

**Table 1:** Hydrogen-bond geometry (Å, °)

*D*—H⋯*A*	*D*—H	H⋯*A*	*D*⋯*A*	*D*—H⋯*A*
C9—H9*B*⋯*Cg*^i^	0.97	2.99	3.646 (4)	126
C14—H14*A*⋯O4*A*^ii^	0.96	2.57	3.504 (3)	165
C15—H15*C*⋯O4*B*^iii^	0.96	2.54	3.343 (5)	142
C16—H16*A*⋯O4*A*^iv^	0.96	2.39	3.333 (3)	168
C16—H16*A*⋯O4*A*^iv^	0.96	2.39	3.333 (3)	168

Symmetry codes: (i) 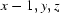; (ii) 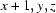; (iii) 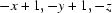; (iv) 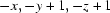. *Cg* is the centroid of C2–C7 benzene ring.

**Fig. 1.**
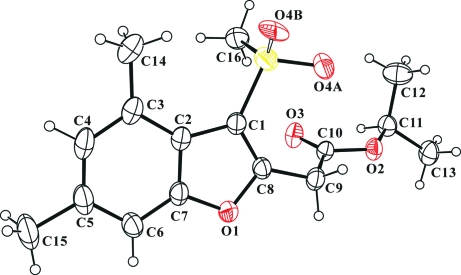
The molecular structure of the title compound, showing displacement ellipsoids drawn at the 30% probability level.

**Fig. 2.**
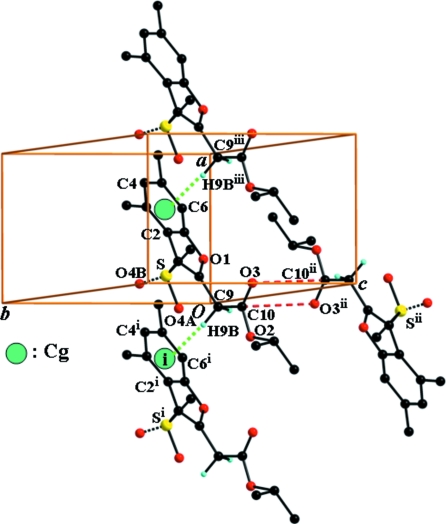
C—H···π and C···O interactions (dotted lines) in the title compound. Cg denotes ring centroid. [Symmetry code: (i) x - 1, y, z; (ii) -x, -y, -z + 1; (iii) x + 1, y, z.]

## supplementary crystallographic information

### Comment

This work is related to our previous communications on the synthesis and structure of alkyl 2-(3-methylsulfinyl-1-benzofuran-2-yl)acetate analogues, *viz*. ethyl 2-(5-methyl-3-methylsulfinyl-1-benzofuran-2-yl)acetate (Choi *et al.*, 2007) and isopropyl 2-(5-methyl-3-methylsulfinyl-1-benzofuran-2-yl) acetate (Choi *et al.*, 2008). Here we report the crystal structure of the title compound, isopropyl 2-(4,6-dimethyl-3-methylsulfinyl-1-benzofuran-2-yl) acetate (Fig. 1).

The benzofuran unit is essentially planar, with a mean deviation of 0.011 (2) Å from the least-squares plane defined by the nine constituent atoms. The O4 atom of the methylsulfinyl group is disordered over two positions with site-occupancy factors fixed at 0.9 (for atom O4A) and 0.1 (for atom O4B) in Fig. 1. The molecular packing (Fig. 2) is stabilized by C—H···π interactions between the hydrogen of 9-methylene group and a benzene ring of benzofuran unit, with a C9—H9B···*Cg*^i^ separation of 2.99 Å (Fig. 2 & Table 1) (*Cg* is the centroid of C2–C7 benzene ring, symmetry code as in Fig. 2). The molecular packing is further stabilized by intermolecular C—H···O hydrogen bonds (Table 1). In addition, the crystal packing exhibits a type-II carbonyl–carbonyl interaction (Allen *et al.*, 1998), with C10···O3 ^ii^ and O3···C10^ii^ distance of 3.295 (2) Å (symmetry code as in Fig. 2).

### Experimental

77% 3-chloroperoxybenzoic acid (314 mg, 1.40 mmol) was added in small portions to a stirred solution of isopropyl 2-(4,6-dimethyl-3-methylsulfanyl-1-benzofuran-2-yl)acetate (380 mg, 1.30 mmol) in dichloromethane (40 ml) at 273 K. After being stirred for 3 h at room temperature, the mixture was washed with saturated sodium bicarbonate solution and the organic layer was separated, dried over magnesium sulfate, filtered and concentrated in vacuum. The residue was purified by column chromatography (hexane-ethylcetate, 1:2 *v*/*v*) to afford the title compound as a colorless solid [yield 84%, m.p. 357–358 K; *R*_f_ = 0.77 (hexane-ethyl acetate, 1;2 *v*/*v*)]. Single crystals suitable for X-ray diffraction were prepared by evaporation of a solution of the title compound in acetone at room temperature. Spectroscopic analysis: ^1^H NMR (CDCl_3_, 400 MHz) δ 1.26 (d, J = 6.24 Hz, 6H), 2.42 (s, 3H), 2.62 (s, 3H), 2.99 (s, 3H), 4.34 (s, 2H), 5.00–5.08 (m, 1H), 6.93 (s, 1H), 7.13 (s, 1H); EI—MS 308 [*M*^+^].

### Refinement

All H atoms were geometrically positioned and refined using a riding model, with C—H = 0.98 (methine), 0.93 (aromatic), 0.97 (methylene), and 0.96 Å (methyl) H atoms, respectively, and with *U*_iso_(H) = 1.2Ueq(C) (aromatic, methylene, methine), and 1.5Ueq(C) (methyl) H atoms. The O4 atom of the methylsulfinyl group is disordered over two positions with site-occupancy factors fixed at 0.90 (O4A) and 0.10 (O4B) in final refinement. The displacement ellipsoids of O4B was restrained using command ISOR (0.01) and both O4 atoms were restrained usig command DELU. The distances of S—O4 (A & B) were restrained using command SADI (0.001).

### Crystal data

**Table:**

C_16_H_20_O_4_S	*Z* = 2
*M**_r_* = 308.38	*F*(000) = 328
Triclinic, *P*1	*D*_x_ = 1.296 Mg m^−^^3^
Hall symbol: -P 1	Mo *K*α radiation, λ = 0.71073 Å
*a* = 6.308 (1) Å	Cell parameters from 2459 reflections
*b* = 11.340 (2) Å	θ = 2.4–28.0°
*c* = 11.506 (2) Å	µ = 0.22 mm^−^^1^
α = 81.403 (3)°	*T* = 298 K
β = 77.205 (3)°	Block, colorless
γ = 83.167 (4)°	0.40 × 0.30 × 0.10 mm
*V* = 790.4 (2) Å^3^	

### Data collection

**Table:**

Bruker SMART CCD diffractometer	2270 reflections with *I* > 2σ(*I*)
Radiation source: fine-focus sealed tube	*R*_int_ = 0.044
graphite	θ_max_ = 25.0°, θ_min_ = 2.4°
Detector resolution: 10.0 pixels mm^-1^	*h* = −6→7
φ and ω scans	*k* = −8→13
4195 measured reflections	*l* = −13→13
2760 independent reflections	

### Refinement

**Table:**

Refinement on *F*^2^	Primary atom site location: structure-invariant direct methods
Least-squares matrix: full	Secondary atom site location: difference Fourier map
*R*[*F*^2^ > 2σ(*F*^2^)] = 0.043	Hydrogen site location: difference Fourier map
*wR*(*F*^2^) = 0.126	H-atom parameters constrained
*S* = 1.06	*w* = 1/[σ^2^(*F*_o_^2^) + (0.0676*P*)^2^ + 0.1659*P*] where *P* = (*F*_o_^2^ + 2*F*_c_^2^)/3
2760 reflections	(Δ/σ)_max_ < 0.001
202 parameters	Δρ_max_ = 0.40 e Å^−^^3^
7 restraints	Δρ_min_ = −0.27 e Å^−^^3^

### Special details

**Table:**

Geometry. All e.s.d.'s (except the e.s.d. in the dihedral angle between two l.s. planes) are estimated using the full covariance matrix. The cell e.s.d.'s are taken into account individually in the estimation of e.s.d.'s in distances, angles and torsion angles; correlations between e.s.d.'s in cell parameters are only used when they are defined by crystal symmetry. An approximate (isotropic) treatment of cell e.s.d.'s is used for estimating e.s.d.'s involving l.s. planes.
Refinement. Refinement of *F*^2^ against ALL reflections. The weighted *R*-factor *wR* and goodness of fit *S* are based on *F*^2^, conventional *R*-factors *R* are based on *F*, with *F* set to zero for negative *F*^2^. The threshold expression of *F*^2^ > σ(*F*^2^) is used only for calculating *R*-factors(gt) *etc*. and is not relevant to the choice of reflections for refinement. *R*-factors based on *F*^2^ are statistically about twice as large as those based on *F*, and *R*- factors based on ALL data will be even larger.

### Fractional atomic coordinates and isotropic or equivalent isotropic displacement parameters (Å^2^)

**Table:**

	*x*	*y*	*z*	*U*_iso_*/*U*_eq_	Occ. (<1)
S	0.14112 (9)	0.43525 (5)	0.31658 (6)	0.0639 (2)	
O1	0.2743 (2)	0.14311 (12)	0.15511 (12)	0.0528 (4)	
O2	−0.2895 (2)	0.15214 (13)	0.49041 (11)	0.0503 (4)	
O3	0.0716 (2)	0.13761 (14)	0.48320 (14)	0.0612 (4)	
O4A	−0.0855 (3)	0.41447 (18)	0.37312 (18)	0.0742 (5)	0.90
O4B	0.082 (3)	0.5555 (7)	0.2602 (15)	0.086 (5)	0.10
C1	0.2493 (3)	0.31729 (18)	0.22999 (17)	0.0471 (5)	
C2	0.4558 (3)	0.30687 (18)	0.14521 (17)	0.0480 (5)	
C3	0.6322 (3)	0.3783 (2)	0.10060 (19)	0.0573 (5)	
C4	0.8036 (4)	0.3295 (3)	0.0188 (2)	0.0697 (7)	
H4	0.9231	0.3739	−0.0118	0.084*	
C5	0.8091 (4)	0.2190 (3)	−0.0207 (2)	0.0705 (7)	
C6	0.6334 (3)	0.1514 (2)	0.0214 (2)	0.0634 (6)	
H6	0.6307	0.0778	−0.0044	0.076*	
C7	0.4621 (3)	0.19809 (19)	0.10351 (17)	0.0504 (5)	
C8	0.1482 (3)	0.21824 (18)	0.23284 (17)	0.0478 (5)	
C9	−0.0638 (3)	0.1747 (2)	0.30051 (18)	0.0538 (5)	
H9A	−0.0826	0.1005	0.2732	0.065*	
H9B	−0.1814	0.2330	0.2825	0.065*	
C10	−0.0808 (3)	0.15375 (17)	0.43450 (18)	0.0461 (4)	
C11	−0.3372 (3)	0.13159 (19)	0.62208 (17)	0.0523 (5)	
H11	−0.2126	0.0850	0.6498	0.063*	
C12	−0.3741 (5)	0.2506 (2)	0.6681 (2)	0.0836 (8)	
H12A	−0.4951	0.2967	0.6402	0.100*	
H12B	−0.2455	0.2927	0.6393	0.100*	
H12C	−0.4055	0.2389	0.7543	0.100*	
C13	−0.5315 (4)	0.0596 (2)	0.6574 (2)	0.0671 (6)	
H13A	−0.4967	−0.0144	0.6234	0.080*	
H13B	−0.6524	0.1041	0.6280	0.080*	
H13C	−0.5699	0.0430	0.7434	0.080*	
C14	0.6363 (4)	0.4994 (2)	0.1375 (2)	0.0748 (7)	
H14A	0.7017	0.4908	0.2065	0.112*	
H14B	0.4898	0.5360	0.1571	0.112*	
H14C	0.7199	0.5488	0.0725	0.112*	
C15	1.0074 (4)	0.1738 (4)	−0.1096 (3)	0.1018 (11)	
H15A	0.9817	0.0989	−0.1311	0.153*	
H15B	1.1329	0.1626	−0.0735	0.153*	
H15C	1.0327	0.2313	−0.1804	0.153*	
C16	0.3028 (4)	0.3962 (2)	0.4279 (2)	0.0661 (6)	
H16A	0.2627	0.4515	0.4863	0.099*	
H16B	0.4543	0.3996	0.3905	0.099*	
H16C	0.2784	0.3166	0.4669	0.099*	

### Atomic displacement parameters (Å^2^)

**Table:**

	*U*^11^	*U*^22^	*U*^33^	*U*^12^	*U*^13^	*U*^23^
S	0.0584 (4)	0.0639 (4)	0.0712 (4)	0.0072 (3)	−0.0154 (3)	−0.0215 (3)
O1	0.0453 (8)	0.0637 (9)	0.0494 (8)	−0.0128 (6)	0.0000 (6)	−0.0156 (6)
O2	0.0364 (7)	0.0699 (9)	0.0424 (7)	−0.0089 (6)	−0.0025 (6)	−0.0057 (6)
O3	0.0408 (8)	0.0785 (10)	0.0620 (9)	−0.0078 (7)	−0.0115 (7)	0.0015 (7)
O4A	0.0467 (10)	0.0921 (14)	0.0844 (13)	0.0064 (9)	−0.0059 (9)	−0.0346 (10)
O4B	0.088 (9)	0.086 (8)	0.088 (9)	−0.014 (7)	−0.012 (7)	−0.027 (7)
C1	0.0400 (10)	0.0573 (12)	0.0441 (10)	−0.0055 (8)	−0.0075 (8)	−0.0074 (8)
C2	0.0408 (10)	0.0620 (12)	0.0406 (10)	−0.0089 (9)	−0.0087 (8)	−0.0012 (9)
C3	0.0474 (11)	0.0727 (14)	0.0510 (12)	−0.0190 (10)	−0.0111 (9)	0.0069 (10)
C4	0.0452 (12)	0.108 (2)	0.0529 (13)	−0.0264 (12)	−0.0032 (10)	0.0042 (13)
C5	0.0440 (12)	0.116 (2)	0.0469 (12)	−0.0070 (12)	0.0000 (10)	−0.0109 (13)
C6	0.0504 (12)	0.0878 (17)	0.0509 (12)	−0.0058 (11)	−0.0005 (10)	−0.0202 (11)
C7	0.0400 (10)	0.0681 (13)	0.0420 (10)	−0.0106 (9)	−0.0043 (8)	−0.0051 (9)
C8	0.0392 (10)	0.0619 (12)	0.0413 (10)	−0.0055 (9)	−0.0033 (8)	−0.0102 (9)
C9	0.0402 (10)	0.0725 (14)	0.0492 (11)	−0.0129 (9)	−0.0039 (9)	−0.0116 (10)
C10	0.0363 (10)	0.0491 (11)	0.0521 (11)	−0.0078 (8)	−0.0054 (8)	−0.0065 (8)
C11	0.0484 (11)	0.0654 (13)	0.0408 (10)	−0.0062 (9)	−0.0055 (8)	−0.0045 (9)
C12	0.118 (2)	0.0786 (18)	0.0577 (15)	−0.0077 (16)	−0.0186 (15)	−0.0185 (13)
C13	0.0555 (13)	0.0899 (17)	0.0500 (12)	−0.0156 (12)	0.0016 (10)	−0.0021 (11)
C14	0.0683 (15)	0.0767 (17)	0.0809 (17)	−0.0326 (13)	−0.0162 (13)	0.0071 (13)
C15	0.0542 (15)	0.168 (3)	0.0741 (18)	−0.0068 (17)	0.0147 (13)	−0.0319 (19)
C16	0.0635 (14)	0.0744 (15)	0.0670 (14)	−0.0040 (11)	−0.0174 (11)	−0.0257 (12)

### Geometric parameters (Å, °)

**Table:**

C10—O3^i^	3.295 (2)	C8—C9	1.490 (3)
S—O4B	1.463 (2)	C9—C10	1.506 (3)
S—O4A	1.464 (2)	C9—H9A	0.9700
S—C1	1.769 (2)	C9—H9B	0.9700
S—C16	1.783 (2)	C11—C12	1.497 (3)
O1—C7	1.378 (2)	C11—C13	1.501 (3)
O1—C8	1.382 (2)	C11—H11	0.9800
O2—C10	1.332 (2)	C12—H12A	0.9600
O2—C11	1.466 (2)	C12—H12B	0.9600
O3—C10	1.199 (2)	C12—H12C	0.9600
C1—C8	1.349 (3)	C13—H13A	0.9600
C1—C2	1.448 (3)	C13—H13B	0.9600
C2—C7	1.382 (3)	C13—H13C	0.9600
C2—C3	1.412 (3)	C14—H14A	0.9600
C3—C4	1.387 (3)	C14—H14B	0.9600
C3—C14	1.502 (4)	C14—H14C	0.9600
C4—C5	1.389 (4)	C15—H15A	0.9600
C4—H4	0.9300	C15—H15B	0.9600
C5—C6	1.380 (3)	C15—H15C	0.9600
C5—C15	1.521 (3)	C16—H16A	0.9600
C6—C7	1.379 (3)	C16—H16B	0.9600
C6—H6	0.9300	C16—H16C	0.9600
			
O4B—S—O4A	93.2 (8)	O2—C10—C9	109.90 (16)
O4B—S—C1	121.5 (7)	O3—C10—O3^i^	79.52 (13)
O4A—S—C1	107.56 (10)	O2—C10—O3^i^	83.17 (11)
O4B—S—C16	127.3 (7)	C9—C10—O3^i^	107.38 (13)
O4A—S—C16	108.31 (12)	O2—C11—C12	108.31 (17)
C1—S—C16	97.44 (10)	O2—C11—C13	105.86 (16)
C7—O1—C8	106.29 (15)	C12—C11—C13	114.3 (2)
C10—O2—C11	117.50 (15)	O2—C11—H11	109.4
C8—C1—C2	107.41 (17)	C12—C11—H11	109.4
C8—C1—S	124.50 (15)	C13—C11—H11	109.4
C2—C1—S	128.08 (16)	C11—C12—H12A	109.5
C7—C2—C3	119.24 (19)	C11—C12—H12B	109.5
C7—C2—C1	104.92 (17)	H12A—C12—H12B	109.5
C3—C2—C1	135.8 (2)	C11—C12—H12C	109.5
C4—C3—C2	115.5 (2)	H12A—C12—H12C	109.5
C4—C3—C14	121.5 (2)	H12B—C12—H12C	109.5
C2—C3—C14	123.0 (2)	C11—C13—H13A	109.5
C3—C4—C5	124.5 (2)	C11—C13—H13B	109.5
C3—C4—H4	117.7	H13A—C13—H13B	109.5
C5—C4—H4	117.7	C11—C13—H13C	109.5
C6—C5—C4	119.4 (2)	H13A—C13—H13C	109.5
C6—C5—C15	120.6 (3)	H13B—C13—H13C	109.5
C4—C5—C15	120.0 (3)	C3—C14—H14A	109.5
C7—C6—C5	116.9 (2)	C3—C14—H14B	109.5
C7—C6—H6	121.6	H14A—C14—H14B	109.5
C5—C6—H6	121.6	C3—C14—H14C	109.5
O1—C7—C6	124.8 (2)	H14A—C14—H14C	109.5
O1—C7—C2	110.74 (16)	H14B—C14—H14C	109.5
C6—C7—C2	124.4 (2)	C5—C15—H15A	109.5
C1—C8—O1	110.62 (16)	C5—C15—H15B	109.5
C1—C8—C9	134.01 (18)	H15A—C15—H15B	109.5
O1—C8—C9	115.37 (17)	C5—C15—H15C	109.5
C8—C9—C10	113.33 (16)	H15A—C15—H15C	109.5
C8—C9—H9A	108.9	H15B—C15—H15C	109.5
C10—C9—H9A	108.9	S—C16—H16A	109.5
C8—C9—H9B	108.9	S—C16—H16B	109.5
C10—C9—H9B	108.9	H16A—C16—H16B	109.5
H9A—C9—H9B	107.7	S—C16—H16C	109.5
O3—C10—O2	125.19 (18)	H16A—C16—H16C	109.5
O3—C10—C9	124.88 (17)	H16B—C16—H16C	109.5
			
O4B—S—C1—C8	−114.6 (9)	C5—C6—C7—O1	−179.9 (2)
O4A—S—C1—C8	−9.3 (2)	C5—C6—C7—C2	0.2 (3)
C16—S—C1—C8	102.6 (2)	C3—C2—C7—O1	−178.61 (17)
O4B—S—C1—C2	65.6 (9)	C1—C2—C7—O1	0.9 (2)
O4A—S—C1—C2	170.83 (18)	C3—C2—C7—C6	1.4 (3)
C16—S—C1—C2	−77.2 (2)	C1—C2—C7—C6	−179.1 (2)
C8—C1—C2—C7	−0.5 (2)	C2—C1—C8—O1	−0.2 (2)
S—C1—C2—C7	179.39 (15)	S—C1—C8—O1	179.98 (13)
C8—C1—C2—C3	179.0 (2)	C2—C1—C8—C9	179.6 (2)
S—C1—C2—C3	−1.2 (4)	S—C1—C8—C9	−0.2 (3)
C7—C2—C3—C4	−1.7 (3)	C7—O1—C8—C1	0.7 (2)
C1—C2—C3—C4	179.0 (2)	C7—O1—C8—C9	−179.11 (17)
C7—C2—C3—C14	177.9 (2)	C1—C8—C9—C10	−62.5 (3)
C1—C2—C3—C14	−1.5 (4)	O1—C8—C9—C10	117.28 (19)
C2—C3—C4—C5	0.6 (3)	C11—O2—C10—O3	1.4 (3)
C14—C3—C4—C5	−179.0 (2)	C11—O2—C10—C9	179.47 (16)
C3—C4—C5—C6	0.9 (4)	C11—O2—C10—O3^i^	73.42 (14)
C3—C4—C5—C15	−179.2 (2)	C8—C9—C10—O3	−21.3 (3)
C4—C5—C6—C7	−1.3 (3)	C8—C9—C10—O2	160.60 (17)
C15—C5—C6—C7	178.8 (2)	C8—C9—C10—O3^i^	−110.50 (16)
C8—O1—C7—C6	179.0 (2)	C10—O2—C11—C12	92.1 (2)
C8—O1—C7—C2	−1.0 (2)	C10—O2—C11—C13	−144.89 (18)

Symmetry codes: (i) −*x*, −*y*, −*z*+1.

### Hydrogen-bond geometry (Å, °)

**Table:**

*D*—H···*A*	*D*—H	H···*A*	*D*···*A*	*D*—H···*A*
C9—H9B···Cg^ii^	0.97	2.99	3.646 (4)	126
C14—H14A···O4A^iii^	0.96	2.57	3.504 (3)	165
C15—H15C···O4B^iv^	0.96	2.54	3.343 (5)	142
C16—H16A···O4A^v^	0.96	2.39	3.333 (3)	168
C16—H16A···O4A^v^	0.96	2.39	3.333 (3)	168

Symmetry codes: (ii) *x*−1, *y*, *z*; (iii) *x*+1, *y*, *z*; (iv) −*x*+1, −*y*+1, −*z*; (v) −*x*, −*y*+1, −*z*+1.
